# Identifying and mapping measures of medication safety during transfer of care in a digital era: a scoping literature review

**DOI:** 10.1136/bmjqs-2022-015859

**Published:** 2023-11-03

**Authors:** Catherine Leon, Helen Hogan, Yogini H Jani

**Affiliations:** 1 Department of Health Services Research and Policy, London School of Hygiene & Tropical Medicine, London, UK; 2 Department of Practice and Policy, University College London School of Pharmacy, London, UK; 3 Centre for Medicines Optimisation Research and Education, University College London Hospitals NHS Foundation Trust, London, UK

**Keywords:** Healthcare quality improvement, Medical error, measurement/epidemiology, Medication safety, Patient safety, Transitions in care

## Abstract

**Background:**

Measures to evaluate high-risk medication safety during transfers of care should span different safety dimensions across all components of these transfers and reflect outcomes and opportunities for proactive safety management.

**Objectives:**

To scope measures currently used to evaluate safety interventions targeting insulin, anticoagulants and other high-risk medications during transfers of care and evaluate their comprehensiveness as a portfolio.

**Methods:**

Embase, Medline, Cochrane and CINAHL databases were searched using scoping methodology for studies evaluating the safety of insulin, anticoagulants and other high-risk medications during transfer of care. Measures identified were extracted into a spreadsheet, collated and mapped against three frameworks: (1) ‘Key Components of an Ideal Transfer of Care’, (2) work systems, processes and outcomes and (3) whether measures captured past harms, events in real time or areas of concern. The potential for digital health systems to support proactive measures was explored.

**Results:**

Thirty-five studies were reviewed with 162 measures in use. Once collated, 29 discrete categories of measures were identified. Most were outcome measures such as adverse events. Process measures included communication and issue identification and resolution. Clinic enrolment was the only work system measure. Twenty-four measures captured past harm (eg, adverse events) and six indicated future risk (eg, patient feedback for organisations). Two real-time measures alerted healthcare professionals to risks using digital systems. No measures were of advance care planning or enlisting support.

**Conclusion:**

The measures identified are insufficient for a comprehensive portfolio to assess safety of key medications during transfer of care. Further measures are required to reflect all components of transfers of care and capture the work system factors contributing to outcomes in order to support proactive intervention to reduce unwanted variation and prevent adverse outcomes. Advances in digital technology and its employment within integrated care provide opportunities for the development of such measures.

**WHAT IS ALREADY KNOWN ON THIS TOPIC**

- High-risk medications such as insulin and anticoagulants can cause harm if issues occur during transfer of care. Studies to improve the safety of these processes have used many different measures to determine whether these interventions had an impact.

**WHAT THIS STUDY ADDS**

- This study identifies a range of measures currently used and assesses their comprehensiveness as a portfolio for evaluating the safety of high-risk medications during transfer of care. It identifies where gaps in measurement exist.

**HOW THIS STUDY MIGHT AFFECT RESEARCH, PRACTICE OR POLICY**

- The measurement gaps found provide an opportunity to develop indicators which reflect healthcare complexity, real-time risks and can be used to improve safety proactively. Digital systems in integrated care present new opportunities for comprehensive measurement approaches through real-time data collection and analysis spanning the whole patient pathway.

## Introduction

Keeping patients safe from harm is a central goal of health services. Despite decades of international effort, improvement is still required.^1^ Medication errors are a leading cause of avoidable harm.^2^ During transfers of care (ToC) patients move between healthcare settings and are at greater risk of medication-related harm.^3^ Adverse events following ToC from hospital to home are common.^4^ Nearly 40% of medication errors occur during care transfer, and 20% of those errors are estimated to cause harm.^5^ Between 30% and 70% of people experience an error with their medications after ToC.^6 7^ Multiple processes must be undertaken to ensure that people’s medications are managed safely during this period. Common barriers to safe transfer include poor communication, inadequate patient, family and/or carer involvement and insufficient provision of supporting services.^8^ Failures in these processes or activities can lead to incorrect medications or doses, causing harm from underdosing or overdosing, or through accidental provision of an incorrect medication. Where support systems are not identified and arranged, patients may not be able to obtain or take their medications at all.^3 8 9^ The WHO set a Global Safety Challenge in 2017 to reduce severe, avoidable medication-related harm by 50% over 5 years, with ‘Medication Safety in Transitions of Care’ identified as a key focus for improvement.^3^ In developing this safety challenge, a comprehensive review of the literature was performed and the WHO provided some suggested measures that could be used to evaluate the impact of improvement programmes; however, these do not constitute a detailed measurement portfolio.^3^ Other systematic reviews of safety during ToC focus on potential strategies for improvement rather than methods for evaluating success.^10 11^

High-risk medications (HRMs) carry a greater risk of harm when errors occur.^12 13^ Errors are not necessarily more common with these medications, but the consequences of errors are potentially life threatening. People taking these medications have a heightened risk of medication-related harm during or following ToC.^3 4^ Commonly recognised HRMs include insulin, anticoagulants, opioids, sedatives, concentrated electrolytes, anti-infectives and chemotherapeutic agents.^14^ These medications continue to cause serious harm despite focused safety improvement work. Insulin and anticoagulants are common HRMs used to treat long-term conditions across all care settings in adults of all ages and are associated with risks during ToC.^15–19^ In England, targeted patient safety alerts have aimed to improve access to up-to-date dosing information and related blood tests for HRMs (insulin and anticoagulants) during ToC through patient-held records.^15 17^

**Figure 1:**
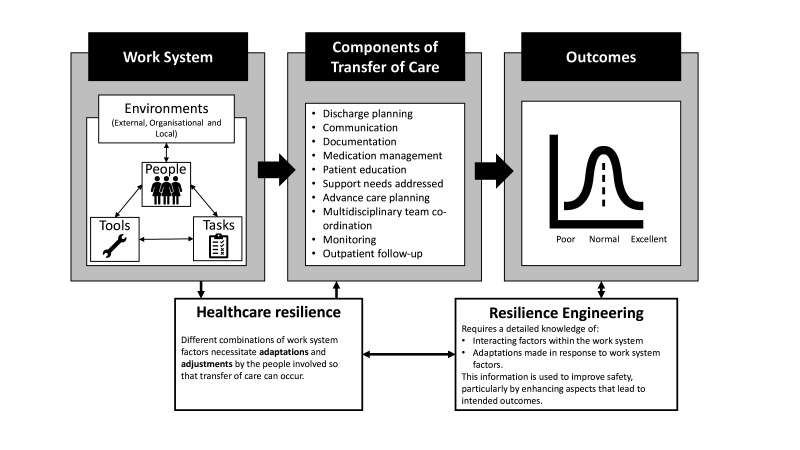
Healthcare resilience and resilience engineering and how these influence the components of transfers of care (ToC) and outcomes.^26 47^

To improve safety, it is important to define what safety is. Traditionally, it has been considered as the absence of harm, and improvement efforts have focused on learning from past adverse events.^20^ This assumes that poor outcomes are caused by discernible, measurable factors that can be addressed and eliminated to prevent recurrence.^20 21^ It is now understood that healthcare takes place in a complex, dynamic system requiring work to be adapted and adjusted in the face of individual circumstances.^20 22 23^ The healthcare work system is commonly understood to include people (patients, informal carers, healthcare professionals and other staff), equipment, tasks and the environments in which the healthcare is provided (both locally and more widely).^24–27^ The adaptations and adjustments that are necessary to maintain high-quality care in the face of variation and challenges are known as healthcare resilience.^28^ Resilient adaptations can be made by individuals or at higher levels, such as in a ward or across an organisation.^28–30^ Using this perspective, safety can be conceptualised as the capacity of the system to enable things to go well.^31^ Resilience engineering is the study of the work system and healthcare resilience to develop mechanisms to promote successful outcomes; see figure 1 for an illustration of these concepts in relation to ToC.

Measurement and monitoring are required to assess whether safety is improving. As safety cannot be measured directly, measures are used as indicators of safety. Carefully developed portfolios of indicators are required to ensure comprehensive measurement covering multiple aspects of safety including different perspectives of staff, organisations and patients.^32^ Traditionally, retrospective (lagging) measures of harm have been employed to provide intelligence around safety and allow comparison over time.^33^ Assessing safety, characterised as an emergent phenomenon within a complex work system,^34^ requires measures that are collected prospectively (leading) or in real or near-real time which identify areas of variation in work system factors and tasks that make up the processes of care. Capturing variation provides insight into both areas of potential risk where intervention can be made to prevent harm and also system resilience by revealing how challenges are being resolved, and how conditions for successful outcomes are created.^31 35^ Resilience engineering approaches can be used to identify these indicators,^21 36^ and the advent of digital technology provides opportunities for their collection.

Digital technology is critical for the development of a broader array of safety measures. It enables rapid, targeted sharing of information to promote proactive interventions to improve safety. Advances, such as the introduction of artificial intelligence tools and natural language processing, promise efficient analysis of data gathered across multiple care settings.^37 38^ They will facilitate searching for indicators of safety across the vast quantities of textual information held within health records and feedback forums to provide rapid insights around staff and patient experience and outcomes.^39–41^ Integration of data from patient portals and wearable technology, such as fitness trackers and continuous glucose monitors, can enable remote monitoring and identification of risks in near real time.^42–45^

The focus of this scoping review was to identify the range of measures that are currently being used to evaluate the safety of insulin, anticoagulants and other HRMs during ToC. The objectives were to establish how well existing measures reflect a comprehensive indicator portfolio for the safety of these medications at ToC, whether they reflect systems, processes or outcomes and whether these may be used for both ongoing monitoring of safety and proactive intervention to prevent harm. The secondary aim was to assess the adaptability of the measures for digitisation.

## Methods

Embase, Medline, Cochrane and CINAHL databases were searched using a scoping methodology.^46^ This approach allowed the systematic identification and mapping of measures related to safety improvement across a broad literature employing disparate approaches to the evaluation of safety improvement interventions in varying contexts. These measures were then compared, and gaps identified. Selected databases were deemed most likely to contain studies relating to medication safety improvement. Search terms included transfer*, medic* reconciliation, transition, transfer, and insulin*, anticoag*, anti-coag* and high-risk medic*. Full details are included in online supplemental file 1. Searches were performed using the full databases including all years available. Results were limited to English language and human studies. A protocol can be found in online supplemental file 2.

10.1136/bmjqs-2022-015859.supp1 Supplementary data

10.1136/bmjqs-2022-015859.supp2 Supplementary data

Duplicate references were removed, and titles and abstracts were screened according to the following criteria. To be included, the study had to relate to adults of 18 years or over, involve a ToC (including between wards within a single organisation), focus on anticoagulants, insulin or HRMs as a group and involve evaluation of an intervention designed to improve the safety or quality of the medications involved. Studies where no interventions were performed or where the impact of an intervention on safety or quality was not evaluated were excluded. All measures used to determine the effectiveness of a safety intervention were included provided there was sufficient information to replicate the measure. Randomised and non-randomised controlled trials, before and after studies, interrupted time-series studies, historically controlled studies and research protocols detailing clearly planned measures were included. Case studies, case reports, unpublished studies, opinion pieces and cross-sectional studies were excluded. Conference abstracts were included providing there was sufficient detail to understand the measures used to evaluate the intervention.

The full text of papers that met the inclusion criteria was scrutinised to identify the intervention, whether it targeted anticoagulants, insulin or HRMs as a group, the type of ToC and whether electronic health systems were used, and in what manner. Measures were extracted from the studies and grouped into inductively developed categories according to the overarching aim of the measure. Three frameworks were used to map the measures of the different activities involved in ToC, the extent to which work systems, processes and outcomes were each measured and the spread of these measures in terms of whether they were lagging, leading or real time. By using three frameworks, the different aspects of complexity, potential for proactive measurement and across the care transition, could be explored.

The first framework, the Key Components of an Ideal Transfer of Care (KCoIToC), is a theoretical model capturing the different activities such as discharge planning or communication required to perform a successful ToC developed by Burke *et al.* 47 The second framework, Systems Engineering Initiative for Patient Safety (SEIPS), was used to determine whether identified measures provided insight into work systems, processes or outcomes. SEIPS is a human factor-based framework ‘nested within’^27^ Donabedian’s quality model of structure, process and outcomes.^24–27^ It was created as a tool to support the in-depth understanding of health and care structures (termed work systems) and to identify barriers and facilitators of safety within them. Processes are defined as a combination of tasks and the work system components required to perform them.^24^ Variation in processes which drive outcomes stems from the interactions between work system components and tasks. For each process measure, where relevant, the different work system factors that contribute to that process were considered. For example, the process of communication between inpatient and outpatient clinicians involves several different work system factors including people, tasks and tools. The people involved are the patient whose care is being discussed, the inpatient clinician and the receiving outpatient clinician. The tasks include performing the communication (verbal or written), receiving the communication and documentation. The tools required could include communication devices such as telephones, electronic health systems or emails. By considering the range of factors contributing to the process, potential targets for additional measures can be found. These can be used to provide more detailed insight into process variation. The timing of the measures in terms of whether they were lagging, leading or real time^35^ was used as the third framework. Considering the measures in this way allows the spread of reactive and proactive measures to be assessed.

Finally, studies were examined to determine whether measures were obtained from digital health systems (DHS) in real time or if they had the potential to be obtained in this way. Real-time measures might be derived from digital systems that identify if a key task has not been completed and alert staff of required action, those that collect real-time information from patients via patient-held digital health records or alerting systems related to extreme blood test results.

One author extracted the data, developed the categories and mapped the measures to the frameworks. The mapping was discussed with the other two authors and consensus reached in cases of disagreement. The measures and the mapping were reviewed at intervals, and any uncertainties were considered and addressed as a team. The team was composed of three healthcare professionals, two with a hospital background and one with a background in primary care. This provided insight into the activities being measured, particularly in mapping according to the SEIPS framework.^24–27^

## Results

**Figure 2:**
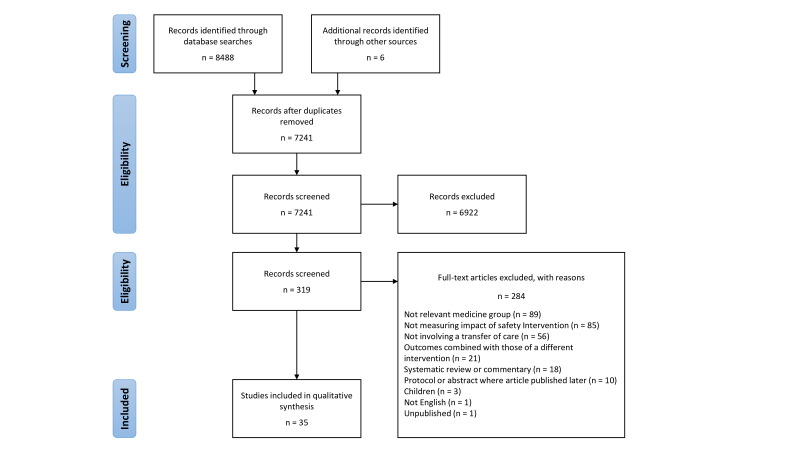
Literature screening process.

A total of 8488 studies were identified from the four databases, with a total of 7235 unique studies (see figure 2). An additional six articles were identified by scrutinising the references of included articles.

After applying the inclusion and exclusion criteria, 35 studies were eligible. They were published between 2011 and 2022. Most studies took place in the USA (25), with four from Australia and one each from Brazil, China, France, Italy, Saudi Arabia and Spain. The studies principally focused on anticoagulation (21). The remaining studies explored HRMs as a group of medications (10) and insulin (4). Twenty-five were original research reports and ten were abstracts from conference proceedings. See table 1 for an overview of each study. A more detailed table is provided as online supplemental table 1 listing the measures used in each study.

10.1136/bmjqs-2022-015859.supp3 Supplementary data

**Table 1:** References and key information

Year	Author	Article/abstract	Study design; number of participants	Medication type	Intervention to improve safety	Care transition
2011	Avanzini *et al* 57	Article	Observational study; 142	Insulin	Standardised protocol	Intensive cardiac care unit to general ward
2011	Nordenholz *et al* 58	Abstract	Cohort study; 106	Anticoagulant	Clinical care pathway	Emergency department to primary care
2011	Reger *et al* 59	Article	Observational study; 207	Anticoagulant	Discharge pathway	Hospital to primary care
2011	Schillig *et al* 60	Article	Randomised controlled trial; 500	Anticoagulant	Pharmacist involvement	Hospital to primary care
2011	Stafford *et al* 61	Article	Cohort study; 268	Anticoagulant	Pharmacist involvement	Hospital to primary care
2012	Falana *et al* 62	Abstract	Cohort study; 88	Anticoagulant	Pharmacist involvement	Hospital to outpatient clinic
2013	Martin III *et al* 63	Article	Cohort study; not defined	High-risk medications	Pharmacist involvement	Hospital to primary care
2014	Falconieri *et al* 64	Article	Cohort study; 32	Anticoagulant	Transfer of care programme	Emergency department to primary care
2014	Martins *et al* 65	Abstract	Randomised clinical trial; 280	Anticoagulant	Outpatient clinic	Outpatient clinic to primary care
2015	Padron and Miyares^66^	Article	Cohort study; 409	Anticoagulant	Anticoagulation stewardship programme	Hospital to outpatient care
2015	Dunn *et al* 67	Article	Cohort study; 797	Anticoagulant	Information pack	Hospital to outpatient clinic
2015	Quach *et al* 68	Abstract	Randomised controlled trial; 307	High-risk medications	Medication reconciliation	Primary care to the emergency department
2015	Yilmaz *et al* 69	Abstract	Randomised controlled trial; protocol only	High-risk medications	Medication reconciliation and discharge counselling	Hospital to primary care
2016	Ha *et al* 70	Article	Cohort study; 109	Anticoagulant	Standardised protocol	Hospital to primary care
2017	Bryant *et al* 71	Abstract	Retrospective observational analysis; 220	Anticoagulant	Pharmacist involvement	Emergency department to primary care
2017	Castelli *et al* 72	Article	Randomised controlled trial; 25	Anticoagulant	Information pack for patients	Hospital to primary care
2017	Chamoun *et al* 73	Article	Cohort study; 206	Anticoagulant	Standardised protocol	Hospital to primary care
2017	Wei *et al* 49	Article	Randomised controlled trial; 28	Insulin	Remote glucose monitoring	Hospital to primary care
2017	Zdyb *et al* 74	Article	Retrospective record analysis; 85	Anticoagulant	Counselling and education	Emergency department to primary care
2018	Herges *et al* 75	Article	Retrospective record analysis; 1004	High-risk medications	Pharmacist involvement	Hospital to primary care
2019	Dempsey *et al* 76	Abstract	Observational study; 247	High-risk medications	Pharmacist involvement	Hospital to primary care
2019	Pyrlis *et al* 77	Article	Randomised controlled trial; 105	Insulin	Transition diabetes team	Hospital to primary care
2020	Kapoor *et al* 56	Article	Randomised controlled trial; 162	Anticoagulant	Pharmacist involvement	Hospital to primary care
2020	Liang *et al* 78	Article	Randomised controlled trial; 152	Anticoagulant	Pharmacist involvement	Hospital to primary care
2020	Lim *et al* 79	Article	Retrospective case series; 120	Anticoagulant	Outpatient clinic	Emergency department to outpatient clinic
2020	Tyedin *et al* 80	Article	Cohort study; 238	Anticoagulant	Pharmacist involvement	Hospital to primary care
2020	Andre *et al* 81	Abstract	Observational study; 162	Anticoagulant	Medication reconciliation	Primary care to hospital
2022	Bakey and Nguyen^82^	Article	Cohort study; 58	Anticoagulant	Pharmacist involvement	Emergency department to primary care
2021	Bawazeer *et al* 83	Abstract	Randomised controlled trial; 107	High-risk medications	Medication reconciliation, counselling, follow-up	Hospital to primary care
2021	DeSancho *et al* 84	Article	Quality improvement; 409	Anticoagulant	Counselling and education	Hospital to primary care
2021	Gurwitz *et al* 85	Article	Randomised controlled trial; 361	High-risk medications	Pharmacist involvement	Hospital to primary care
2021	Kane-Gill *et al* 48	Article	Quality improvement; 2127	High-risk medications	Pharmacist involvement	Primary care to nursing home
2021	Magny-Normilus *et al* 86	Article	Randomised controlled trial; 180	Insulin	Discharge intervention	Hospital to primary care
2021	Zabrosky *et al* 87	Abstract	Quality improvement; 218	High-risk medications	Standardised protocols for transfer of care	Hospital to primary care
2022	Lázaro Cebas *et al* 88	Article	Cohort study; 589	High-risk medications	Pharmacist involvement	Hospital to primary care

A total of 162 measures were collated and mapped. There were 15 measures identified from studies relating to insulin, 38 for HRMs and 109 from studies relating to anticoagulants. Eight measures were excluded as they were not described in sufficient detail to understand how they were used, for example, ‘laboratory ordering practices’ and ‘medication stopped’ with no further information.

Measures were grouped into 29 inductively developed categories. These were adverse events (thrombosis, bleeding, death, hypoglycaemia or hyperglycaemia, readmission rates) (n=61), time in therapeutic range (n=14), medication-related problems (numbers identified (n=12), their potential for harm (n=3), recommendations made (n=2) and recommendations accepted (n=2)), adherence (the extent to which patients follow a medication regimen agreed with their prescribing healthcare professional) (n=7), assessment of patient knowledge, understanding and beliefs (n=7), patient satisfaction (n=6), education and counselling (n=3), outpatient appointments (time to follow-up (n=4), appointment attendance (n=4), enrolment into clinic or appointment made (n=3)), time to reach therapeutic range (n=2), pharmacist time (n=2), protocol adherence (n=4), availability of medicines confirmed (n=2), patients with blood test within 10 days (n=1), therapeutic drug monitoring performed (n=1), baseline laboratory information available (n=1), time outside therapeutic range (n=1), cost of intervention (n=1), documentation of information in discharge letter (n=4), pharmacist coordination documented (n=1), clinician satisfaction (n=1), medication titration frequency (n=1), inadequate follow-up arrangements (n=1), documented communication (inpatient-to-outpatient (n=1) and inpatient-to-anticoagulation clinic (n=1)) and intravenous access obtained (n=1).

### Measures identified

Most measures identified were lagging, outcome measures of adverse events and aspects of blood test monitoring. There were process measures that included both leading and lagging indicators. Although many potential specific work system factors were referred to in papers, these were not measured. Only one work system measure (the rate of appointments booked) was identified in the studies.

By far, the most frequently used category of measures were the rates of adverse events such as bleeding or thrombosis (with anticoagulants) or hypoglycaemia (insulin) as well as rates of readmissions and mortality. These were lagging, outcome indicators and related to the ‘Medication Safety’ component of the KCoIToC. Other medication safety measures included the number of issues identified or rectified and rates of adherence to protocols, all of which were lagging measures counted retrospectively. ‘Educating patients to promote self-management’ was the second most frequently measured component with measures of patient satisfaction and medication adherence falling into this category. These were often lagging measures for the patients for whom the healthcare experience had been completed but could be used as a leading measure by the organisation. Monitoring and managing symptoms after discharge was another component with many lagging outcome measures and one real-time measure identified. These included aspects of blood test monitoring, particularly for insulin and anticoagulants. Documentation and communication measures were lagging and of processes. They related to the ‘Complete communication of information component’. Availability of baseline bloods was measured in one study and related to the component of ‘Availability, timeliness, clarity and organisation of information’. Aspects of ‘Co-ordinating care among team members’ were measured through documentation of pharmacist involvement and clinician satisfaction. These were process and outcome measures, which were all lagging. The ‘Outpatient follow-up’ component included measures of appointment attendance (a lagging process measure) and the time taken for the follow-up to occur (a lagging outcome measure). No measures were found that covered the components ‘Advance Care Planning’ or ‘Enlisting the help of social and community supports’.

Studies that aimed to improve the safety of anticoagulants and HRMs as a group often focused on measuring specific aspects of prescribing quality and accuracy along with interventions made by healthcare professionals to improve safety. Follow-up arrangements were measured in several studies. Three studies measured aspects of efficiency such as the time involved to undertake the intervention and the cost of the intervention. One study measured staff experience.

Table 2 summarises the range of measures identified, mapped according to KCoIToC component, SEIPS and timing.

**Table 2:** Measures identified categorised according to the KCoIToC processes and mapped according to SEIPS, their timing and the potential for real-time use

KCoIToC component	Measures associated with KCoIToC components (SEIPS work system elements involved (people, tasks, tools, environments))	SEIPS framework measured/timing (lagging, leading, real time)	Potential for real time using digital health systems
Discharge planning	Enrolment into clinic/outpatient appointment made^60 84 87^ Tasks: booking appointment, documenting appointment	Work system/leading	Documentation and alert.*
	Access obtained for home injections of high-risk medication^87^ People: patient, staff Task: performing cannulation Tool: cannulation equipment	Outcome/lagging	Documentation and alert.
	Medication availability confirmed^76 87^ People: patient and/or carer, staff Task: determining medication availability Tools: medication, telephone, computer	Process/leading	Documentation and alert.
	Percentage of inadequate warfarin follow-up arrangements^63^ People: patient and healthcare professional Tasks: identify follow-up requirements, arrange follow-up Tools: digital health system, telephone, computer, diary	Process/lagging	†
Complete communication of information	Documented inpatient-to-outpatient provider contact^60^ People: healthcare professionals Task: documentation Tool: form of communication (paper or electronic)	Process/lagging	Documentation and alert.
	Documented inpatient-to-anticoagulation clinic communication^60^ People: healthcare professionals Task: documentation Tool: form of communication (paper or electronic)	Process/lagging	Documentation and alert.
	Information in discharge letter^70 80 82 87^ People: healthcare professionals Task: documentation Tool: form of communication (paper or electronic)	Process/lagging	Documentation and alert.
Availability, timeliness, clarity and organisation of information	Baseline laboratory information available^87^ People: patient, staff, laboratory staff Tasks: request, take, analyse and report blood test Tools: blood test result (electronic or paper report), patient record	Process/lagging	Alert if baseline blood test results are not available when prescription written.
Medication safety	Adverse events (hypoglycaemia or hyperglycaemia, venous thromboembolism, readmissions, death, cardiovascular events)^49 57–62 64–66 69 70 72–80 82–84 86–88^	Outcome/lagging	Some, for example, abnormal blood tests.
	Medications managed according to protocol^71 72 74 87^ People: patient, prescriber, pharmacy Task: prescribing Tools: medication, prescription, protocol	Process/lagging	†
	Medication discrepancies, errors or issues identified^63 69 75 76 80–83 85 87^ People: patient, prescriber, healthcare professional reviewing medications Task: medication review Tools: medications, references (eg, medication information leaflets, reference books)	Process/lagging and real time	Documentation with targeted alert to prompt review.
	Rate of recommendations agreed^63 75^ People: patient, staff (recommendation maker and prescriber) Tasks: prescribing, documentation	Process/lagging	†
	Medication safety recommendations made^71 75^ People: patient, staff (recommendation maker and prescriber) Tasks: prescribing, documentation	Process/lagging	Documentation and alert.
	Impact of interventions to optimise medications^48 68 81^	Outcome/lagging	†
Educating patients to promote self-management	Measures of adherence^64 69 72 74 84 86^ People: patient (and caregiver) Task: taking medication Tools: medication, packaging, compliance aids (eg, tablet cutters)	Process/leading	Through patient-owned digital method, for example, access to their electronic health record or smartphone application.
	Patient satisfaction^56 64 69 72 77 83^	Outcome/lagging for patient Leading for organisation	
	Provision of education and counselling^71 82 87^ People: patient, healthcare professional Task: providing education Tools: information leaflets, medication charts	Process/leading	Partially—tasks (eg, education) can be documented and highlighted if outstanding.
	Assessment of patient knowledge, understanding and beliefs^56 72 78 81 84^ People: patient, assessor Task: assessment of knowledge Tool: assessment template/quiz	Process/leading	†
Enlisting social and community supports			
Advance care planning			
Coordinating care among team members	Percentage of patients with pharmacist coordination documented^59^ People: patient, pharmacist, multidisciplinary team Tasks: ‘co-ordination’ tasks, documentation Tool: patient records	Process/lagging	Documentation and alert.
	Pharmacist time per patient^59 87^	Outcome/lagging	†
	Cost of intervention^88^	Outcome/lagging	†
	Clinician satisfaction^67^	Outcome/lagging	†
Monitoring and managing symptoms after transfer	Time in therapeutic range^49 57 61 65 66 70 73 77 78 83 86^	Outcome/lagging and real time	Viewed within patient record.
	Time outside therapeutic range^78^	Outcome/lagging	†
	Time to reach therapeutic range^67 73^	Outcome/lagging	†
	Therapeutic drug monitoring performed^87^ People: patient, staff, laboratory staff Tasks: request, take, analyse, report blood test Tools: blood test equipment, laboratory equipment to analyse, blood test result (electronic or paper report)	Process/lagging	Documentation and alert.
	Percentage of international normalised ratio taken within 10 days of transfer of care^67^ People: patient, staff, laboratory staff Tasks: request, take, analyse, report blood test Tools: blood test equipment, laboratory equipment to analyse, blood test result (electronic or paper report)	Process/lagging	Documentation and alert.
Outpatient follow-up	Clinic appointment attendance^64 66 67^ People: patient, staff Tasks: book, communicate and attend appointment	Process/lagging	Documentation and alert.
	Time to follow-up^60 64 71 83^	Outcome/lagging	†

*Documentation of a specific task with an associated alert targeted to relevant staff prompting action if that task remains outstanding.

†Not applicable.

KCoIToC, Key Components of an Ideal Transfer of Care; SEIPS, Systems Engineering Initiative for Patient Safety.

### DHS use

Only two studies collected real-time (or near real-time) measures and used these to adjust care. Kane-Gill *et al* 48 alerted healthcare professionals of patients at risk of harm via an electronic patient record to facilitate early intervention. Wei *et al* 49 used an internet-based portal to monitor study participants’ blood sugar levels, and where significantly abnormal, the results were reviewed and insulin doses adjusted. Although not described in any studies except Kane-Gill *et al*,^48^ many measures had the potential to use DHS to alert staff in real time where tasks have not been documented and therefore may be overdue for completion, as shown in table 2. There were additional lost opportunities to use specific test results and patient-documented adherence information in a real-time manner.

## Discussion

Although many measures were identified they did not constitute a comprehensive portfolio for assessing HRM safety during ToC. Measures did not fully represent all components of ToC and were primarily focused on past events. Traditional outcome-based measures were the most used. Although useful for gaining a broad overview of the safety and effectiveness of HRM during ToC, they offer limited insight into where interventions for improvement might be best focused. There were many potential work system factors that could have been measured across studies but there was only evidence of one being measured directly, rates of enrolment to a clinic. Work system factors are key to understanding variation in process measures and ultimately outcomes and providing insight into resilience. This is especially valuable if performance is directly communicated in real time, providing the opportunity for proactive interventions to improve safety.

The KCoIToC are very broad, each consisting of many tasks and influenced by many work system factors. Without a more detailed understanding of each component, the role of adaptations and adjustments in determining outcomes cannot be understood. For example, ‘Co-ordinating care among team members’ would benefit from a comprehensive understanding of how work system factors such as staff and equipment availability impact on outcomes and drive variability in safety. Such an understanding would identify approaches that could strengthen healthcare resilience.^21^

Comprehensive measurement portfolios can support understanding of how good outcomes are maintained despite varying conditions, providing a window of opportunity for proactive care adjustments to avoid harm. Peñaloza *et al* 50 developed five ‘guidelines’ to assess whether indicator frameworks can be used to measure the resilience capacities within the healthcare system and therefore be used to improve safety using resilience engineering.^28^ These guidelines state that measures must provide insight into the resilient adaptations and complexities of healthcare that are contributing to outcomes. Second, measures should be targeted to the relevant individual who needs to act and should be provided in real time. Third, they should support efforts to learn from what is going well in addition to what is unsuccessful. Fourth, the measures should provide insight into trade-offs between safety and other issues, for example, if safety checks are being omitted due to time pressures in a clinic. Finally, the portfolio of measures should evolve as the processes and work changes over time.^50^ Without indicators illuminating the complex, interacting factors of the work system and resilient activities performed during ToC of HRMs, the measures obtained from this literature scoping review cannot yet be used for resilience engineering and enhancing capacity for successful, high-quality care. Incorporating these guidelines when developing measurement portfolios will foster the inclusion of indicators that provide insight into the complexity and resilience of healthcare delivery and the underlying causes of variability linked to safety. This enables exploration of factors that contribute to success and focused interventions to improve safety.

Many safety measures would be amenable to real-time measurement if certain tasks were recorded in the electronic patient record. It is essential that all users are involved in the development and testing of such measures as well as the design of electronic health systems so that capturing the required information is not too burdensome for users (healthcare staff and patients).^32 51^ As digital technologies are advancing, there is great potential for developing new measures taking advantage of these systems. For example, wearable technology, smartphone applications and data warehouses could all potentially be valuable sources of data if used within appropriate governance arrangements. Machine learning and natural language processing also provide opportunities for identifying measures within unstructured narrative data that have previously been too labour intensive for routine use, for example, from medical notes, compliments and complaints.

Patients and their caregivers contribute greatly to the safety of ToC, adapting their actions to prevent and overcome issues.^52 53^ There were very few measures that accounted for the active role that patients perform in the ToC process. Patient contributions are becoming ever more possible with ongoing developments to digital patient-held records and healthcare tools.^54^ Within the measurement category of ‘Educating the patient to promote self-management’, measures included elements of patient involvement, for example, adherence. The patient is key in this process; however, many factors influence their decision to adhere to the medication regimen such as their core beliefs about taking medications, their risk and benefit analysis of the medications and lifestyle factors.^55^ Many of these factors are not reflected by the indicators identified in this literature review, with only Kapoor *et al* assessing aspects of patient’s beliefs regarding anticoagulation.^56^ Evaluating the contribution of patients and the resilience activities they perform will provide valuable ways to include these essential aspects of safety. This will result in a more holistic measurement approach.

### Strengths and limitations

The literature review used a systematic approach with clearly defined concepts to explore and identify a wide range of indicators. Inclusion of insulin and anticoagulants along with HRMs in general expanded the breadth of measures identified. Most interventions in the review were aimed at improving discharge from hospital to primary care, with other aspects of ToC less well represented. There may be additional relevant measures that could be detected by including studies of other HRMs, medication safety in general or other potential contexts for ToC. Components of ToC may also vary between countries which potentially limits wider generalisability. Furthermore, the framework of the KCoIToC is designed to assess the transition from hospital to primary care, although many components remain valid for other ToC. The SEIPS framework is a tool that is designed to highlight the impact of interactions between different factors within the work system, processes and outcomes. The limited detail in the literature did not lend itself to in-depth analysis of interacting work system factors using SEIPS. The authors used their prior knowledge and experience to identify some of these factors, but this was not exhaustive. In developing further measures, a more detailed exploration of the relevant work systems is required.

## Conclusion

This literature review identified a range of measures that can be used as part of a portfolio to evaluate the safety of ToC for people taking anticoagulants, insulin or HRMs. The identified measures were insufficient to provide insight from a resilience engineering perspective. Measures predominantly stemmed from a traditional approach to safety management, providing an overview of general outcomes. There is potential to identify new leading indicators of safety by obtaining a deep understanding of the complex work system interactions and resilience activities that maintain the safety of HRMs during ToC. A comprehensive, patient-centred safety measurement framework for ToC and HRMs should include such leading indicators, targeted in real time to relevant people across care pathways that can enable early intervention. Digital health technology implementation is essential for such an approach.

## Data Availability

All data relevant to the study are included in the article or uploaded as supplementary information.
