# Supplementary material for: Identifying and mapping measures of medication safety during transfer of care in a digital era: a scoping literature review
Source: BMJ Qual Saf. 2023 Nov 3;33(3):173–86. doi: 10.1136/bmjqs-2022-015859 (PMC10894843; doi:10.1136/bmjqs-2022-015859)
Supplement: Supplementary data [file bmjqs-2022-015859supp001.pdf]

Literature search terms

Medline

Database: Ovid MEDLINE(R) ALL <1946 to May 19, 2022>
Search Strategy:
----

1 (transfer* adj3 care).mp. (3892)
2 (escalation* adj3 care).mp. (558)
3 medic* discrepanc*.mp. (454)
4 medic* reconciliation.mp. (2508)
5 (transition* adj3 care).mp. (10410)
6 (hospital adj3 discharge).mp. (43449)
7 admission.mp. (235405)
8 Patient transfer/ (9417)
9 or/1-8 (290357)

10 insulin*.mp. or insulin/ or insulin passport*.mp. (453098)
11 (anticoag* or anti-coag*).mp. or anticoagulant agent/ (146420)
12 (("high alert" or "high risk") adj2 (medicine* or medication* or drug*)).mp. (1780)
13 (safe* or harm* or risk* or error* or hazard*).mp. (4559838)
14 (("high alert" or "high risk") adj2 (medicine* or medication* or drug*) adj4 (safe* or harm* or risk* or error* or hazard*)).mp. (1658)
15 ((insulin* or insulin passport*) adj4 (safe* or harm* or risk* or error* or hazard*)).mp. (7702)
16 ((anticoag* or anti-coag*) adj4 (safe* or harm* or risk* or error* or hazard*)).mp. (6653)
17 or/14-16 (15961)

18 9 and 17 (634)
19 limit 18 to (english language and humans) (480)

Embase

Database: Embase Classic+Embase <1947 to 2022 May 19>
Search Strategy:
----

1 (transfer* adj3 care).mp. (7396)
2 (escalation* adj3 care).mp. (1240)
3 medic* discrepanc*.mp. (924)
4 medic* reconciliation.mp. (4382)
5 (transition* adj3 care).mp. (17795)
6 (hospital adj3 discharge).mp. (185175)
7 admission.mp. (536983)
8 Patient transfer/ (30946)
9 or/1-8 (714538)

10 insulin*.mp. or insulin/ or insulin passport*.mp. (922269)
11 (anticoag* or anti-coag*).mp. or anticoagulant agent/ (282900)
12 (("high alert" or "high risk") adj2 (medicine* or medication* or drug*)).mp. (3224)
13 (safe* or harm* or risk* or error* or hazard*).mp. (7041191)
14 (("high alert" or "high risk") adj2 (medicine* or medication* or drug*) adj4 (safe* or harm* or risk* or error* or hazard*)).mp. (2971)
15 ((insulin* or insulin passport*) adj4 (safe* or harm* or risk* or error* or hazard*)).mp. (11952)

16 ((anticoag* or anti-coag*) adj4 (safe* or harm* or risk* or error* or hazard*)).mp. (11555)
17 or/14-16 (26323)

18 9 and 17 (1763)
19 limit 18 to (human and english language) (1631)

Cochrane

Search Name: May22
Date Run: 20/05/2022 16:20:48

ID Search Hits

1. ((care near/1 model*)):ti,ab,kw OR ((model* near/1 service delivery)):ti,ab,kw OR ((model* near/1 (healthcare or health care or health-care))):ti,ab,kw OR ((transform* near/1 (service* or care))):ti,ab,kw (Word variations have been searched) 2641
2. MeSH descriptor: [Patient Transfer] explode all trees 166
3. (transfer near/3 care):ti,ab,kw OR (escalation* near/3 care):ti,ab,kw OR (medic* discrepanc*):ti,ab,kw OR (medic* reconciliation):ti,ab,kw OR (transition* near/3 care):ti,ab,kw 2754
4. #1 or #2 or #3 5410
5. ((safe* or harm* or risk* or error* or hazard*) near/4 (insulin* or anti-coag* or anticoag*)) 4690
6. ((safe* or harm* or risk* or error* or hazard*) near/4 ("high risk medicine" or "high risk medication" or "high risk medicines" or "high risk medications")) 70
7. ((safe* or harm* or risk* or error* or hazard*) near/4 ("high risk drug" or "high alert drug" or "high risk drugs" or "high alert drugs")) 37
8. ((safe* or harm* or risk* or error* or hazard*) near/4 ("high alert medicine" or "high alert medication" or "high alert medicines" or "high alert medications")) 6
9. #5 or #6 or #7 or #8 4796
10. #4 and #9 in Trials 38

Cinahl

MY EBSCOhost

Friday, May 20, 2022 2:10:37 PM

#	Query	Limiters/Expanders	Last Run Via	Results
S52	S35 AND S51	Expanders - Apply equivalent subjects Search modes - Boolean/Phrase	Interface - EBSCOhost Research Databases Search Screen - Advanced Search Database - CINAHL Complete	Display
S51	S50 n4 S49	Expanders - Apply equivalent subjects Search modes - Boolean/Phrase	Interface - EBSCOhost Research Databases Search Screen - Advanced Search Database - CINAHL Complete	Display
S50	S39 OR S44	Expanders - Apply equivalent subjects Search modes - Boolean/Phrase	Interface - EBSCOhost Research Databases Search Screen - Advanced Search Database - CINAHL Complete	Display
S49	S45 OR S46 OR S47 OR S48	Expanders - Apply equivalent subjects Search modes - Boolean/Phrase	Interface - EBSCOhost Research Databases Search Screen - Advanced Search Database - CINAHL Complete	Display
S48	errors or mistakes or incidents or adverse events	Expanders - Apply equivalent subjects Search modes - Boolean/Phrase	Interface - EBSCOhost Research Databases Search Screen - Advanced Search Database - CINAHL Complete	Display
S47	hazard*	Expanders - Apply equivalent subjects Search modes - Boolean/Phrase	Interface - EBSCOhost Research Databases Search Screen - Advanced Search Database - CINAHL Complete	Display

S46	harm	Expanders - Apply equivalent subjects<br>Search modes - Boolean/Phrase	Interface - EBSCOhost Research Databases<br>Search Screen - Advanced Search<br>Database - CINAHL Complete	Display
S45	safety or danger or risks	Expanders - Apply equivalent subjects<br>Search modes - Boolean/Phrase	Interface - EBSCOhost Research Databases<br>Search Screen - Advanced Search<br>Database - CINAHL Complete	Display
S44	S40 OR S41 OR S42 OR S43	Expanders - Apply equivalent subjects<br>Search modes - Boolean/Phrase	Interface - EBSCOhost Research Databases<br>Search Screen - Advanced Search<br>Database - CINAHL Complete	Display
S43	high alert medicine	Expanders - Apply equivalent subjects<br>Search modes - Boolean/Phrase	Interface - EBSCOhost Research Databases<br>Search Screen - Advanced Search<br>Database - CINAHL Complete	Display
S42	high alert medication	Expanders - Apply equivalent subjects<br>Search modes - Boolean/Phrase	Interface - EBSCOhost Research Databases<br>Search Screen - Advanced Search<br>Database - CINAHL Complete	Display
S41	high risk medicine	Expanders - Apply equivalent subjects<br>Search modes - Boolean/Phrase	Interface - EBSCOhost Research Databases<br>Search Screen - Advanced Search<br>Database - CINAHL Complete	Display
S40	high risk medication	Expanders - Apply equivalent subjects<br>Search modes - Boolean/Phrase	Interface - EBSCOhost Research Databases<br>Search Screen - Advanced Search<br>Database - CINAHL Complete	Display

S39	S36 OR S37 OR S38	Expanders - Apply equivalent subjects<br>Search modes - Boolean/Phrase	Interface - EBSCOhost Research Databases<br>Search Screen - Advanced Search<br>Database - CINAHL Complete	Display
S38	anti-coag*	Expanders - Apply equivalent subjects<br>Search modes - Boolean/Phrase	Interface - EBSCOhost Research Databases<br>Search Screen - Advanced Search<br>Database - CINAHL Complete	Display
S37	anticoagulant therapy or anticoagulants	Expanders - Apply equivalent subjects<br>Search modes - Boolean/Phrase	Interface - EBSCOhost Research Databases<br>Search Screen - Advanced Search<br>Database - CINAHL Complete	Display
S36	insulin	Expanders - Apply equivalent subjects<br>Search modes - Boolean/Phrase	Interface - EBSCOhost Research Databases<br>Search Screen - Advanced Search<br>Database - CINAHL Complete	Display
S35	S27 OR S28 OR S29 OR S30 OR S31 OR S32 OR S33 OR S34	Expanders - Apply equivalent subjects<br>Search modes - Boolean/Phrase	Interface - EBSCOhost Research Databases<br>Search Screen - Advanced Search<br>Database - CINAHL Complete	Display
S34	medication discrepancies	Expanders - Apply equivalent subjects<br>Search modes - Boolean/Phrase	Interface - EBSCOhost Research Databases<br>Search Screen - Advanced Search<br>Database - CINAHL Complete	Display
S33	medication reconciliation	Expanders - Apply equivalent subjects<br>Search modes - Boolean/Phrase	Interface - EBSCOhost Research Databases<br>Search Screen - Advanced Search<br>Database - CINAHL Complete	Display

S32	medicine reconciliation	Expanders - Apply equivalent subjects<br>Search modes - Boolean/Phrase	Interface - EBSCOhost Research Databases<br>Search Screen - Advanced Search<br>Database - CINAHL Complete	Display
S31	hospital discharge	Expanders - Apply equivalent subjects<br>Search modes - Boolean/Phrase	Interface - EBSCOhost Research Databases<br>Search Screen - Advanced Search<br>Database - CINAHL Complete	Display
S30	hospital admissions or hospitalization or hospitalisation or hospital stay	Expanders - Apply equivalent subjects<br>Search modes - Boolean/Phrase	Interface - EBSCOhost Research Databases<br>Search Screen - Advanced Search<br>Database - CINAHL Complete	Display
S29	transition of care	Expanders - Apply equivalent subjects<br>Search modes - Boolean/Phrase	Interface - EBSCOhost Research Databases<br>Search Screen - Advanced Search<br>Database - CINAHL Complete	Display
S28	escalation of care	Expanders - Apply equivalent subjects<br>Search modes - Boolean/Phrase	Interface - EBSCOhost Research Databases<br>Search Screen - Advanced Search<br>Database - CINAHL Complete	Display
S27	transfer of care	Expanders - Apply equivalent subjects<br>Search modes - Boolean/Phrase	Interface - EBSCOhost Research Databases<br>Search Screen - Advanced Search<br>Database - CINAHL Complete	Display
S26	S9 AND S25	Expanders - Apply equivalent subjects<br>Search modes - Boolean/Phrase	Interface - EBSCOhost Research Databases<br>Search Screen - Advanced Search<br>Database - CINAHL Complete	2,062

S25	s24 n4 s23	Expanders - Apply equivalent subjects<br>Search modes - Boolean/Phrase	Interface - EBSCOhost Research Databases<br>Search Screen - Advanced Search<br>Database - CINAHL Complete	41,850
S24	S13 OR S18	Expanders - Apply equivalent subjects<br>Search modes - Boolean/Phrase	Interface - EBSCOhost Research Databases<br>Search Screen - Advanced Search<br>Database - CINAHL Complete	110,092
S23	S19 OR S20 OR S21 OR S22	Expanders - Apply equivalent subjects<br>Search modes - Boolean/Phrase	Interface - EBSCOhost Research Databases<br>Search Screen - Advanced Search<br>Database - CINAHL Complete	1,515,702
S22	errors or mistakes or incidents or adverse events	Expanders - Apply equivalent subjects<br>Search modes - Boolean/Phrase	Interface - EBSCOhost Research Databases<br>Search Screen - Advanced Search<br>Database - CINAHL Complete	219,238
S21	hazard*	Expanders - Apply equivalent subjects<br>Search modes - Boolean/Phrase	Interface - EBSCOhost Research Databases<br>Search Screen - Advanced Search<br>Database - CINAHL Complete	118,537
S20	harm	Expanders - Apply equivalent subjects<br>Search modes - Boolean/Phrase	Interface - EBSCOhost Research Databases<br>Search Screen - Advanced Search<br>Database - CINAHL Complete	42,032
S19	safety or danger or risks	Expanders - Apply equivalent subjects<br>Search modes - Boolean/Phrase	Interface - EBSCOhost Research Databases<br>Search Screen - Advanced Search<br>Database - CINAHL Complete	1,332,654

S18	S14 OR S15 OR S16 OR S17	Expanders - Apply equivalent subjects<br>Search modes - Boolean/Phrase	Interface - EBSCOhost Research Databases<br>Search Screen - Advanced Search<br>Database - CINAHL Complete	559
S17	high alert medicine	Expanders - Apply equivalent subjects<br>Search modes - Boolean/Phrase	Interface - EBSCOhost Research Databases<br>Search Screen - Advanced Search<br>Database - CINAHL Complete	5
S16	high alert medication	Expanders - Apply equivalent subjects<br>Search modes - Boolean/Phrase	Interface - EBSCOhost Research Databases<br>Search Screen - Advanced Search<br>Database - CINAHL Complete	170
S15	high risk medicine	Expanders - Apply equivalent subjects<br>Search modes - Boolean/Phrase	Interface - EBSCOhost Research Databases<br>Search Screen - Advanced Search<br>Database - CINAHL Complete	39
S14	high risk medication	Expanders - Apply equivalent subjects<br>Search modes - Boolean/Phrase	Interface - EBSCOhost Research Databases<br>Search Screen - Advanced Search<br>Database - CINAHL Complete	348
S13	S10 OR S11 OR S12	Expanders - Apply equivalent subjects<br>Search modes - Boolean/Phrase	Interface - EBSCOhost Research Databases<br>Search Screen - Advanced Search<br>Database - CINAHL Complete	109,625
S12	anti-coag*	Expanders - Apply equivalent subjects<br>Search modes - Boolean/Phrase	Interface - EBSCOhost Research Databases<br>Search Screen - Advanced Search<br>Database - CINAHL Complete	417

S11	anticoagulant therapy or anticoagulants	Expanders - Apply equivalent subjects<br>Search modes - Boolean/Phrase	Interface - EBSCOhost Research Databases<br>Search Screen - Advanced Search<br>Database - CINAHL Complete	28,087
S10	insulin	Expanders - Apply equivalent subjects<br>Search modes - Boolean/Phrase	Interface - EBSCOhost Research Databases<br>Search Screen - Advanced Search<br>Database - CINAHL Complete	81,424
S9	S1 OR S2 OR S3 OR S4 OR S5 OR S6 OR S7 OR S8	Expanders - Apply equivalent subjects<br>Search modes - Boolean/Phrase	Interface - EBSCOhost Research Databases<br>Search Screen - Advanced Search<br>Database - CINAHL Complete	147,848
S8	medication discrepancies	Expanders - Apply equivalent subjects<br>Search modes - Boolean/Phrase	Interface - EBSCOhost Research Databases<br>Search Screen - Advanced Search<br>Database - CINAHL Complete	294
S7	medication reconciliation	Expanders - Apply equivalent subjects<br>Search modes - Boolean/Phrase	Interface - EBSCOhost Research Databases<br>Search Screen - Advanced Search<br>Database - CINAHL Complete	2,572
S6	medicine reconciliation	Expanders - Apply equivalent subjects<br>Search modes - Boolean/Phrase	Interface - EBSCOhost Research Databases<br>Search Screen - Advanced Search<br>Database - CINAHL Complete	85
S5	hospital discharge	Expanders - Apply equivalent subjects<br>Search modes - Boolean/Phrase	Interface - EBSCOhost Research Databases<br>Search Screen - Advanced Search<br>Database - CINAHL Complete	14,505

S4	hospital admissions or hospitalization or hospitalisation or hospital stay	Expanders - Apply equivalent subjects<br>Search modes - Boolean/Phrase	Interface - EBSCOhost Research Databases<br>Search Screen - Advanced Search<br>Database - CINAHL Complete	133,984
S3	transition of care	Expanders - Apply equivalent subjects<br>Search modes - Boolean/Phrase	Interface - EBSCOhost Research Databases<br>Search Screen - Advanced Search<br>Database - CINAHL Complete	1,808
S2	escalation of care	Expanders - Apply equivalent subjects<br>Search modes - Boolean/Phrase	Interface - EBSCOhost Research Databases<br>Search Screen - Advanced Search<br>Database - CINAHL Complete	193
S1	transfer of care	Expanders - Apply equivalent subjects<br>Search modes - Boolean/Phrase	Interface - EBSCOhost Research Databases<br>Search Screen - Advanced Search<br>Database - CINAHL Complete	429
