# Supplementary material for: Identifying and mapping measures of medication safety during transfer of care in a digital era: a scoping literature review
Source: BMJ Qual Saf. 2023 Nov 3;33(3):173–86. doi: 10.1136/bmjqs-2022-015859 (PMC10894843; doi:10.1136/bmjqs-2022-015859)
Supplement: Supplementary data [file bmjqs-2022-015859supp002.pdf]

Review Protocol:

Identifying and mapping measures of medication safety during transfer of care in a digital era: A scoping literature review

Background:

When people experience ToC, they are at heightened risk of medication-related harm, particularly from high-risk medications.[1] Anticoagulants and insulin are high-risk medications used long term across all care settings in people of all ages. They have been the focus of safety improvement work, and issues related to ToC are well documented.[2,3]

For successful ToC, multiple activities and processes must be performed. Ten Key Components of an Ideal ToC (KCoIToC) from a hospital to a community setting have been described by Burke et al.[4] They include: discharge planning, complete communication of information, availability, timeliness, clarity and organisation of information, medication safety, educating patients to self-manage, enlisting social and community support, advance care planning, co-ordinating care among team members, monitoring and managing symptoms after discharge and outpatient follow-up.

Safety in health and care is maintained by the resilient adaptations of the people involved in performing the processes of ToC in response to the varying requirements and demands of the work system.[5] The Systems Engineering Initiative for Patient Safety (SEIPS) is a human-factors based framework designed to support visualisation of the health and care system "nested within" Donabedian's quality model of structure, process and outcomes.[6–9] It was created as a tool to support the in-depth understanding of health and care work-systems, and to identify barriers and facilitators of safety within them.

Measurement portfolios also require measures that provide insight into what has happened in the past and monitor this over (lagging measures) in addition to measures that can highlight areas of potential risk (leading indicators).[10] Digital technology allows greater and more immediate access to data, which could facilitate the use of leading indicators.

Objective

The aim of the literature review is to identify measures used to evaluate interventions improve the safety of anticoagulants, insulin, and high-risk medications during or after transfers of care. It aims to evaluate the comprehensiveness of these measures as a measurement portfolio.

The objectives are:

1. To systematically identify studies that evaluated an intervention designed to improve the safety of insulin, anticoagulants or high-risk medications as a group of medications
2. To identify all measures used and to map them according to three frameworks:
   a. How the measures relate to work systems, processes or outcomes using the SEIPS framework.[6]
   b. Whether the measures can provide evidence for the key components of a successful transfer of care according to the framework developed by Burke et al.[4]
   c. Whether the measures were lagging, leading or real-time.[10]
3. To identify any gaps in the measurements when assessed against the three frameworks.
4. To consider whether the measures could be identified in real-time with electronic health systems.

Criteria for inclusion and exclusion of studies

Table 1: Criteria for inclusion and exclusion of studies

	Included	Excluded
**Population**	Must focus on all three of the following:<br>• Patients transferring between care settings (including between wards within a single organisation).<br>• Patients taking anti-coagulants, insulin or high-risk medications in general.	• Focus on care in a single setting, e.g., Intensive Care Units with no reference to the transfer process.<br>• Focus of study not on insulin, anticoagulants, or high-risk medications.
**Construct of interest**	Any measure used to describe the effectiveness of the safety intervention including:<br>• Performance-based<br>• Clinician-reported<br>• Patient-reported	Studies that did not seek to determine whether intervention led to an improvement in safety or quality
**Comparison**	Comparison of patients who received the intervention compared with a control group, including randomised controlled trials, case-control and cohort studies.	Studies with no comparator groups, for example measures developed:<br>• by expert opinion or Delphi consensus.<br>• using population level data.
**Outcomes**	All measures used to assess whether the safety intervention had an impact will be identified from the studies and used for analysis to determine the comprehensiveness of the measures in terms of:<br>• Whether they represent all the essential elements of transfer of care using the Key Components of an Ideal Transfer of Care framework.<br>• Whether they provide insight into the work-system, processes and outcomes using the Systems Engineering Initiative for Patient Safety framework.<br>• Whether they include lagging, leading and real-time measures.<br>The potential for identifying the measure in real-time using electronic health systems will also be examined.	Measures where there is not enough description or detail to understand how these were obtained or calculated.
**Study design**	Primary research studies	Case reports, case reviews, review articles, unpublished studies, opinion pieces, cross-sectional studies.
**Publication date**	No limit	No limit
**Language**	English	Languages other than English

Search strategy for identification of studies

Four databases will be searched to identify relevant studies: Embase, Medline, Cinahl, and Cochrane databases. These are the prominent health and care related databases deemed most likely to contain studies relating to safety in healthcare.

Search terms will include:

- Transfer of care:
  - Transfer of care
  - Transition of care
  - Escalation
  - Medicine/medication discrepancies
  - Discharge
  - Admission
- Safety:
  - Safe
  - Harm
  - Hazard
  - Risk
  - Error
  - Resilience
- High-Risk Medications:
  - High-risk medication/medicine/drug
  - High-alert medicine/medication/drug
  - Insulin
  - Anticoagulants

Study Selection

An initial review of titles from the search results will identify any potentially meeting the inclusion criteria. A second review of the studies against the abstracts will identify any studies that do not meet the inclusion criteria. Where there is uncertainty, the full text of the article will be obtained to confirm. Any articles meeting the inclusion criteria will be selected, and the full text of the study will be obtained. Where articles are excluded, the reason for exclusion will be documented.

Assessment of methodological quality

The type of article will be identified and recorded, for example whether it was an article published in a journal, or an abstract presented at a conference. Methodological quality assessments will not be performed, as the aim of the study is to identify as many measures of improvement for medications during transfer of care as possible.

Method of data extraction

Each study included in the review will be listed in an excel spreadsheet. For each article, the full text will be read line-by-line and the following will be recorded:

- Citation (author and year of publication)
- Geographical location
- Type of study (design and publication type)
- Number of participants
- Intervention undertaken to improve safety
- Medication involved
- Type of transfer of care being investigated

- Measures used to evaluate the intervention
- Use of digital health systems in identifying, calculating or sharing the measurement data

Data synthesis

The author, CL, will review each measure and consider where they can be grouped together into a broader category, for example different types of adverse events. Each category of measure will then be mapped against three different frameworks in a table, using the framework synthesis approach.

The first framework is the Key Components of an ideal Transfer of Care.[4] This framework lists 10 stages of a discharge that must be completed for that transfer to be successful. By mapping against this framework, the measures will be assessed to understand whether they represent all the activities key to safe transfer. Each measure will be considered as to which component it best represents.

The second framework is the Systems Engineering Initiative for Patient Safety (SEIPS).[6–9] This conceptualises healthcare as taking place in a work system comprising of people, their environments, the tasks performed, and tools used. Processes are performed, and outcomes are influenced by the combination and interactions between the components of the work system and their impact on processes. The measures will be categorized as to whether they are providing insight into work systems, processes, or outcomes. Where possible, the author will use her knowledge and experience to consider what work system factors may influence the measure and list these.

Finally, the measures will be assessed to determine whether they measure past events (lagging indicators), provide an indication of whether an event may occur in the future (leading indicator), or provide real-time data about relevant measures.[10]

The results will be displayed in a table showing how the measures map across different frameworks, and where there are gaps and opportunities for new measures to be developed.

At each stage of data synthesis, the preliminary results will be shared and discussed with the co-authors (categorisation and mapping against each framework and consideration of digital use). The categorisation and mapping will be reviewed, and any ambiguities or challenges will be discussed and considered. The mapping and categorisation will then be agreed by all authors. This process will be repeated at regular intervals as the data synthesis progresses. The three authors are all healthcare professionals, two have a hospital-based background and one with a background in primary care. This provides insight into the context of the studies and measures identified and some aspects of the work-systems.

Results

The search results will use the PRISMA flowchart, detailing the review process and search results, how many articles were excluded and the reasons for exclusion for the exclusion for the articles where the full text was screened.

The included articles will be shared in a table listing the citation, type of study, intervention used, medication involved, the type of transfer of care studied and all the measures used to evaluate the intervention, and digital health system use. Descriptions of the geographical areas will be given in the text.

The measures mapped against the different frameworks will also be presented in a table, and a description of the gaps will be provided in the narrative text.

References:

1 World Health Organization. Medication Safety in Transitions of Care. 2019. http://apps.who.int/bookorders.

2 National Patient Safety Agency. Patient Safety Alert NPSA/2011/PSA003 The adult patient's passport to safer use of insulin. 2011.

3 NPSA. Patient Safety Alert 18: Actions that can make anticoagulant therapy safer (NPSA/2007/18). Published Online First: 2007.http://www.nrls.npsa.nhs.uk/resources/type/alerts/

4 Burke RE, Kripalani S, Vasilevskis EE, *et al.* Moving beyond readmission penalties: creating an ideal process to improve transitional care. *J Hosp Med* 2013;**8**:102–9. doi:10.1002/jhm.1990

5 Wiig S, Aase K, Billett S, *et al.* Defining the boundaries and operational concepts of resilience in the resilience in healthcare research program. *BMC Health Serv Res* 2020;**20**:1–9. doi:10.1186/s12913-020-05224-3

6 Holden RJ, Carayon P. SEIPS 101 and seven simple SEIPS tools. 2021;1–10. doi:10.1136/bmjqs-2020-012538

7 Carayon P, Schoofs Hundt A, Karsh BT, *et al.* Work system design for patient safety: The SEIPS model. *Qual Saf Health Care* 2006;**15**:i50. doi:10.1136/qshc.2005.015842

8 Holden RJ, Carayon P, Gurses AP, *et al.* SEIPS 2.0: a human factors framework for studying and improving the work of healthcare professionals and patients. *Ergonomics* 2013;**56**:1669–86. doi:10.1080/00140139.2013.838643

9 Carayon P, Wooldridge A, Hoonakker P, *et al.* SEIPS 3.0: Human-centered design of the patient journey for patient safety. *Appl Ergon* 2020;**84**:103033. doi:10.1016/j.apergo.2019.103033

10 Vincent C, Burnett S, Carthey J. Safety measurement and monitoring in healthcare: A framework to guide clinical teams and healthcare organisations in maintaining safety. *BMJ Qual Saf* 2014;**23**:670–7. doi:10.1136/bmjqs-2013-002757
