# Supplementary material for: Identifying and mapping measures of medication safety during transfer of care in a digital era: a scoping literature review
Source: BMJ Qual Saf. 2023 Nov 3;33(3):173–86. doi: 10.1136/bmjqs-2022-015859 (PMC10894843; doi:10.1136/bmjqs-2022-015859)
Supplement: Supplementary data [file bmjqs-2022-015859supp003.pdf]

Table 1: References and key information measures used

Year	Author and year	Article or abstract	Medication type	Intervention to improve safety	Care Transition	Electronic Health System Use	Measures used
**2011**	Avanzini et al.[1]	Article	Insulin	Standardised protocol	Intensive cardiac care unit to general ward	Not described	Percentage of blood glucose:<br>• Within a narrow range on the first, second and third days after ToC<br>• Within a wider range after meals on the first, second and third days after ToC<br>Percentage of hypoglycaemia episodes on the first, second and third days after ToC<br>Deaths<br>Rates of main non-lethal cardiovascular complications
**2011**	Nordenholz et al.[2]	Abstract	Anticoagulant	Clinical care pathway	Emergency department to primary care	A standardized electronic order set	Laboratory ordering practices<br>Readmission to an emergency department (ED)<br>Readmission with deep vein thrombosis (DVT)
**2011**	Reger et al.[3]	Article	Anticoagulant	Discharge pathway	Hospital to primary care	Patients identified by scanning computer-based reports.<br>Data collection.	Percentage patients with pharmacist coordination documented<br>Pharmacist time spent per patient<br>Recurrent venous thromboembolism (VTE)<br>Major bleeding
**2011**	Schillig et al.[4]	Article	Anticoagulant	Pharmacist involvement	Hospital to primary care	Not described	Enrolment in anticoagulation clinic<br>Documented inpatient-to-outpatient provider contact<br>Documented inpatient provider-to-anticoagulation clinic communication<br>Patient follow-up with the anticoagulation clinic within five days of discharge<br>Composite of any INR[1] over 5, any episode of major bleeding or development of new

[1] INR stands for international normalised ratio, a blood test used to determine response to vitamin K antagonists (for example warfarin).

Year	Author and year	Article or abstract	Medication type	Intervention to improve safety	Care Transition	Electronic Health System Use	Measures used
							thromboembolic events within 30 days of hospital discharge
**2011**	Stafford et al.[5]	Article	Anticoagulant	Pharmacist involvement	Hospital to primary care	Not described	Major bleeding events within 90 days of discharge<br>Thromboembolic events<br>Rates of death<br>Other adverse events (including minor bleeding)<br>Unplanned hospital readmissions<br>INR:<br>• Control at eight days post-discharge and to day 90<br>• Rates of INR over 4<br>• Rates of INR within, below or above the therapeutic range<br>Rates of persistence with warfarin therapy
**2012**	Falana et al.[6]	Abstract	Anticoagulant	Pharmacist involvement	Hospital to outpatient clinic	Not described	Major or minor bleeding<br>Thromboembolic events<br>INR greater than 5<br>Anticoagulation-related readmissions:<br>• Emergency department (ED) visit<br>• Readmission within 30 days of discharge<br>Successful ToC to the next care provider at discharge.
**2013**	Martin III et al.[7]	Article	High-risk medications	Pharmacist involvement	Hospital to primary care	Pharmacy computer system produced a report identifying patients taking HRMs.	Percentage of discharge orders requiring resolution of:<br>• Medication safety recommendations<br>• Inadequate warfarin follow-up arrangements

Year	Author and year	Article or abstract	Medication type	Intervention to improve safety	Care Transition	Electronic Health System Use	Measures used
							• Unintentional medication changes<br>Rate of physician acceptance of the team's clinical recommendations
**2014**	Falconieri et al.[8]	Article	Anticoagulant	TOC programme	Emergency Department to primary care	Not described	Follow up:<br>• Percentage of patients who attended a follow-up appointment by 30 days<br>• Time to follow-up appointment post-discharge<br>Self-reported anticoagulation adherence<br>Readmission rates<br>Patient satisfaction
**2014**	Martins et al.[9]	Abstract	Anticoagulant	Outpatient clinic	Outpatient clinic to primary care	Not described	Time in therapeutic range<br>Thromboembolic events<br>Number of bleeding events
**2015**	Padron et al. [10]	Article	Anticoagulant	Anticoagulation stewardship program	Hospital to outpatient	Not described	Clinics:<br>• Number of patients seen in clinic<br>• Percentage of patients with therapeutic, subtherapeutic or supratherapeutic INR at clinic appointment<br>• Appointment attendance<br>Adverse events:<br>• Bleeding<br>• Thromboembolic events<br>Readmissions to hospital or ED
**2015**	Dunn et al.[11]	Article	Anticoagulant	Information pack	Hospital to outpatient clinic	Retrospective administrative database review. Electronic health record use not	Change in the frequency of obtaining an INR value within 10 days of discharge<br>Percentage patients attaining a therapeutic INR level within 10 days of discharge

Year	Author and year	Article or abstract	Medication type	Intervention to improve safety	Care Transition	Electronic Health System Use	Measures used
						described.	Clinician satisfaction
**2015**	Quach et al.[12]	Abstract	High-risk medications	Medication reconciliation	Primary care to the Emergency Department	Not described	Potential for errors discovered to cause patient harm or discomfort
**2015**	Yilmaz et al.[13]	Abstract	High-risk medications	Medications reconciliation and discharge counselling	Hospital to primary care	Not described	Adherence<br>Rate of medication reconciliation discrepancies<br>Readmission rates<br>Patient satisfaction
**2016**	Ha et al.[14]	Article	Anticoagulant	Standardised protocol	Hospital to primary care	Patient with medication interactions were identified retrospectively using electronic health record.<br>Standardised data extraction form developed.	Time in therapeutic range<br>Rates of the following during the time of interaction or within 30 days of antimicrobial discontinuation:<br>• Thromboembolic events<br>• Major bleeding events<br>Documentation rates of significant antimicrobial-warfarin interactions
**2017**	Bryant et al.[15]	Abstract	Anticoagulant	Pharmacist involvement	Emergency department to primary care	Not described	Percentage of patients who received appropriate anticoagulation at time of discharge<br>Number of patients with a pharmacist intervention<br>Rates of patient education provided prior to discharge<br>Time to outpatient follow-up

Year	Author and year	Article or abstract	Medication type	Intervention to improve safety	Care Transition	Electronic Health System Use	Measures used
**2017**	Castelli et al.[16]	Article	Anticoagulant	Information pack for patients	Hospital to primary care	A daily report generated to identify patients diagnosed with VTE prescribed rivaroxaban.	Percentage of patients who:<br>• Transitioned to rivaroxaban 20 mg daily on day 22<br>• Had greater than 90% adherence<br>• Stopped rivaroxaban for any reason<br>Adherence<br>Patient understanding of correct dose and timing of medication<br>Overall satisfaction (patient)<br>Rates of:<br>• Minor bleeds<br>• Events that required contacting physician or visiting an emergency department<br>• Recurrent VTE<br>• Death
**2017**	Chamoun et al.[17]	Article	Anticoagulant	Standardised protocol	Hospital to primary care	A report was generated from a patient database, and data collected from electronic healthcare records.	Bleeding:<br>• Rates of bleeding events<br>• INR on day bleeding occurred<br>• Severity of bleeding event<br>• Total number<br>INR:<br>• Composite of changes by 0.5 or more per day or INR greater than 4 during inpatient stay and follow up<br>Percentage of patients achieving a therapeutic stable INR by day 7 and by day 14
**2017**	Wei et al.[18]	Article	Insulin	Remote glucose monitoring	Hospital to primary care	Remote monitoring of glycaemic control using a web-based communication portal.	Mean blood glucose level<br>Exploratory outcomes of hypoglycaemia/hyperglycaemia<br>Insulin titration frequency

Year	Author and year	Article or abstract	Medication type	Intervention to improve safety	Care Transition	Electronic Health System Use	Measures used
**2017**	Zdyb et al.[19]	Article	Anticoagulant	Counselling and education	Emergency department to primary care	Electronic health record used to identify patients requiring interventions.<br>Standardised electronic form for documentation.	Appropriateness of medication dosing<br>Rates of prescription collection<br>If patient had contacted or seen their primary care provider<br>Documented readmission or representation to a hospital within 90 days potentially related to anticoagulation
**2018**	Herges et al.[20]	Article	High-risk medications	Pharmacist involvement	Hospital to primary care	Electronic health record used to calculate risk of patient death or unplanned readmission.<br>Used to calculate percentage of drug therapy problems and medication discrepancies metrics.	Readmission risk at 30, 60 and 180 days<br>Number of drug therapy problem recommendations for all medications and HRMs<br>Percentage of recommendations that were acted on by the clinician within 7 days<br>Number of medication discrepancies for all medications and for HRMs
**2019**	Dempsey et al.[21]	Abstract	High-risk medications	Pharmacist involvement	Hospital to primary care	Not described	Average number of medication discrepancies per patient<br>Number of medication access issues resolved<br>30-day medication related hospital readmissions
**2019**	Pyrlis et al.[22]	Article	Insulin	Transition diabetes team	Hospital to primary care	Not described	Hospital readmissions and emergency department presentations<br>Patient satisfaction<br>Change in HbA1c
**2020**	Kapoor et al.[23]	Article	Anticoagulant	Pharmacist involvement	Hospital to primary care	Nurse reviewed medication list and provided an up-to-date colour version with instructions to the patient by mail.	Quality of care transition using Coleman et al.'s Care Transition Measure (CTM)<br>Patient knowledge regarding anticoagulation, interactions, risks, signs, and symptoms to report to prescriber<br>Anticoagulant beliefs

Year	Author and year	Article or abstract	Medication type	Intervention to improve safety	Care Transition	Electronic Health System Use	Measures used
**2020**	Liang et al.[24]	Article	Anticoagulant	Pharmacist involvement	Hospital to primary care	Not described	Proportions of time within the target INR range during follow-up period<br>Proportions of time within the expanded target range during follow-up period<br>Time spent outside the critical INR range (≤1.5 or ≥ 5.0)<br>Adverse events:<br>• Bleeding<br>• Recurrent thrombosis<br>• Death<br>Readmission<br>Warfarin-related knowledge level
**2020**	Lim et al[25]	Article	Anticoagulant	Outpatient clinic	Emergency department to outpatient clinic	Guidance to clinicians via an electronic clinical decision support tool.	Readmissions<br>Thromboembolic events<br>Bleeding events
**2020**	Tyedin et al.[26]	Article	Anticoagulant	Pharmacist involvement	Hospital to primary care	Electronic health record used by pharmacists to chart and monitor warfarin. Electronic health records used for data collection.	Proportion of patients:<br>• With an INR greater than 5.0<br>• Readmitted relating to anticoagulation<br>• With a complete warfarin dose plan at discharge<br>• With warfarin related errors during admission
**2021**	Andre et al.[27]	Abstract	Anticoagulant	Medication Reconciliation	Primary care to hospital	Not described	Frequency and type of reconciliation discrepancies at admission and discharge<br>Patient knowledge<br>Medication discrepancies rated for severity

Year	Author and year	Article or abstract	Medication type	Intervention to improve safety	Care Transition	Electronic Health System Use	Measures used
**2021**	Bakey et al.[28]	Article	Anticoagulant	Pharmacist involvement	Emergency department to primary care	EHS used to identify eligible patients and document pharmacist recommendations.	Rates of issues relating to care components:<br>• Anticoagulation medication errors at discharge<br>• Patient counselling on anticoagulation<br>• Anticoagulation prescription at discharge<br>Adverse events:<br>• ED or hospital admission for bleeding within 30 days<br>• ED or hospital admission for VTE within 30 days
**2021**	Bawazeer et al.[29]	Abstract	High-risk medications	Medication Reconciliation, counselling and follow up	Hospital to primary care	EHS used to identify patients on insulin and/or warfarin and for data collection	Adverse events:<br>• Readmission rate within 30 days of discharge<br>• Time to first unplanned health care utilization<br>Time to the first outpatient clinic visit<br>Disease-specific parameters (glycosylated haemoglobin (HbA1C) and INR<br>Number of medication-related problems identified during the reconciliation stage<br>Patient satisfaction with the service
**2021**	DeSancho et al.[30]	Journal	Anticoagulant	Counselling and education	Hospital to primary care	Not described	Scheduled follow up appointment<br>Re-admission rates<br>Adverse events:<br>• Recurrent thrombosis<br>• Bleeding events<br>Adherence<br>Anticoagulant recall errors:<br>• Dose<br>• Dose frequency

Year	Author and year	Article or abstract	Medication type	Intervention to improve safety	Care Transition	Electronic Health System Use	Measures used
**2021**	Gurwitz et al.[31]	Article	High-risk medications	Pharmacist involvement	Hospital to primary care	Communication with primary care team through the EHS relating to medication safety. Data collection.	Number of adverse drug-related incidents<br>Clinically important medication errors
**2021**	Kane-Gill et al.[32]	Article	High-risk medications	Pharmacist involvement	Primary care to nursing home	Electronic clinical surveillance system highlighting medication risks.	Patient care recommendations evaluated by degree of harm prevented
**2021**	Magny-Normilus et al.[33]	Article	Insulin	Discharge intervention	Hospital to primary care	Patients identified by scanning EHS reports. Data collected using hospital's clinical data repository.	Adherence<br>Monitoring:<br>• Glycaemic control - change in A1c 60 to 120 days after discharge compared with the A1c in the 90 days before or at the time of index hospitalization<br>• Proportion of monitored patient-days with severe hypoglycaemia (less than 40 mg/dL) within 30 days of discharge<br>Readmissions
**2021**	Zabrosky et al.[34]	Abstract	High-risk medications	Standardised protocols for ToC	Hospital to primary care	Not described	Rate of referral to outpatient follow-up<br>Readmissions<br>Successful TOC protocol completion where evaluation/performed and documentation of following documented:<br>• Baseline laboratory values<br>• Therapeutic drug monitoring<br>• Intravenous access<br>• Drug-drug interactions<br>• Medication availability<br>• Patient counselling on medications<br>• Pharmacist documentation in discharge

Year	Author and year	Article or abstract	Medication type	Intervention to improve safety	Care Transition	Electronic Health System Use	Measures used
							letter<br>Pharmacist time<br>Rate of inappropriate protocol initiation
**2022**	Lázaro Cebas et al.[35]	Article	High-risk medications	Pharmacist involvement	Hospital to primary care	Not described	Readmissions<br>Cost of intervention

References:

1 Avanzini F, Marelli G, Donzelli W, *et al.* Transition from intravenous to subcutaneous insulin: Effectiveness and safety of a standardized protocol and predictors of outcome in patients with acute coronary syndrome. *Diabetes Care*. 2011;34:1445–50.

2 Nordenholz KE, Carlson T, Misky G, *et al.* SAEM Abstracts, Plenary Session. *Academic Emergency Medicine*. 2011:S91. https://doi.org/10.1111/j.1553-2712.2011.01073.x

3 Reger MA, Chapman JL, Lutomski DM, *et al.* Outcomes of a comprehensive, pharmacist-managed injectable anticoagulation discharge program for the prophylaxis and treatment of venous thromboembolism. *J Pharm Technol*. 2011;27:199–205.

4 Schillig J, Kaatz S, Hudson M, *et al.* Clinical and safety impact of an inpatient Pharmacist-Directed anticoagulation service. *J Hosp Med*. 2011;6:322–8.

5 Stafford L, Peterson GM, Bereznicki LRE, *et al.* Clinical outcomes of a collaborative, home-based postdischarge warfarin management service. *Ann Pharmacother*. 2011;45:325–34.

6 Falana O, Flint N, Huynh N, *et al.* Clinical pharmacy anticoagulation service at Rush University Medical Center. *Critical Care Medicine*. San Juan Puerto Rico: Lippincott Williams and Wilkins 2012.

7 Martin III ES, Overstreet RL, Jackson-Khalil LR, *et al.* Implementation of a specialized pharmacy team to monitor high-risk medications during discharge. *Am J Health Syst Pharm*. 2013;70:18–21.

8 Falconieri L, Thomson L, Oettinger G, *et al.* Facilitating anticoagulation for safer transitions: preliminary outcomes from an emergency department deep vein thrombosis discharge program. *Hosp Pract 1995*. 2014;42:16–45.

9 Martins MA, Oliveira JA, Ribeiro DD, *et al.* Abstract 13345: Efficacy and Safety of an Anticoagulation Clinic in Low-income Brazilian Patients With Heart Disease: a Randomized Clinical Trial. *Circulation*. 2014;130. doi: 10.1161/CIRC.130.SUPPL_2.13345

10 Padron M, Miyares MA. Development of an anticoagulation stewardship program at a large tertiary care academic institution. *J Pharm Pract*. 2015;28:93–8.

11 Dunn AS, Shetreat-Klein A, Berman J, *et al.* Improving transitions of care for patients on warfarin: The safe transitions anticoagulation report. *J Hosp Med*. 2015;10:615–8.

12 Quach J, Hua S, Traylor B, *et al.* Reduce medication errors by doing early medication reconciliation in the emergency department. *2015 ACCP Gobal Conference on Clinical Pharmacy*. 2015.

13 Yilmaz Z, Slattery C, Luka B, *et al.* Assessing outcomes of pharmacist driven medication reconciliation and discharge medication counseling of hospitalized high-risk stroke patients. *54th New York State Council of Health-system Pharmacists Annual Assembly*. New York: Journal of pharmacy practice 2015:312–3. https://doi.org/10.1177/0897190015583953

14 Ha NB, Yang K, Hanigan S, *et al.* Impact of a Guideline for the Management of Antimicrobial/Warfarin Interactions in the Inpatient Setting and Across Transition of Care. *Ann Pharmacother*. 2016;50:734–40.

15 Bryant C, McMichael B, Baker JW, *et al.* Assessing pharmacists' influence in ensuring appropriate transitions of care with anticoagulation management: A focus on emergency department discharges. *Pharmacotherapy: The Journal of Human Pharmacology and Drug Therapy*. 2017 ACCP Annual Meeting: John Wiley & Sons, Ltd 2017:e124–238. https://doi.org/10.1002/phar.2052

16 Castelli MR, Saint CA, Nuziale BT, *et al.* Effect of a rivaroxaban patient assistance kit (r-pak) for patients discharged with rivaroxaban: A randomized controlled trial. *Hosp Pharm*. 2017;52:496–501.

17 Chamoun N, Macías CG, Donovan JL, *et al.* Implications of an inpatient warfarin dosing nomogram on safety outcomes post-discharge. *J Thromb Thrombolysis*. 2017;43:454–62.

18 Wei N, Nathan D, Wexler D. Glycemic control after hospital discharge in insulin-treated Type 2 Diabetes: a randomised pilot study of daily remote glucose monitoring. *Physiol Behav*. 2017;176:139–48.

19 Zdyb EG, Courtney DM, Malik S, *et al.* Impact of Discharge Anticoagulation Education by Emergency Department Pharmacists at a Tertiary Academic Medical Center. *J Emerg Med*. 2017;53:896–903.

20 Herges JR, Herges LB, Dierkhising RA, *et al.* Effect of Postdismissal Pharmacist Visits for Patients Using High-Risk Medications. *Mayo Clin Proc Innov Qual Outcomes*. 2018;2:4–9.

21 Dempsey J, Gillis C, De Leon C, *et al.* Improving continuity of care for patients on high-risk medications. *ACMP Managed Care Speciality Pharmacy Annual Meeting*. 2019:S94. www.amcp.org

22 Pyrlis F, Ogrin R, Arthur S, *et al.* Feasibility of using a transition diabetes team to commence injectable therapies postdischarge from a tertiary hospital: A pilot, randomised controlled trial. *BMJ Open*. 2019;9:1–7.

23 Kapoor A, Landyn V, Wagner J, *et al.* Supplying Pharmacist Home Visit and Anticoagulation Professional Consultation during Transition of Care for Patients with Venous Thromboembolism. *J Patient Saf*. 2020;16:E367–75.

24 Liang JB, Lao CK, Tian L, *et al.* Impact of a pharmacist-led education and follow-up service on anticoagulation control and safety outcomes at a tertiary hospital in China: a randomised controlled trial. *Int J Pharm Pract*. 2020;28:97–106.

25 Lim HY, Lambros P, Krishnamoorthi B, *et al.* Outpatient management of deep vein thrombosis using direct oral anticoagulants is safe and efficient. *J Pharm Pract Res*. 2020;50:351–5.

26 Tyedin AE, Taylor SE, Than J, *et al.* Impact of proactive pharmacist-assisted warfarin management using an electronic medication management system in Australian hospitalised patients. *J Pharm Pract Res*. 2020;50:144–51.

27 Andre D, Chatain C, Chaumais M, *et al.* 4CPS-007 Pharmaceutical care as a means of prevention against drug iatrogenesis: case of oral anticoagulants. 2020;27:A50.2-A51.

28 Bakey KH, Nguyen CTN. Impact of a Pharmacist Intervention in the Emergency Department on the Appropriateness of Direct Oral Anticoagulants Prescribed in Venous Thromboembolism Patients. *J Pharm Pract*. 2021;1–7.

29 Bawazeer G, Sales I, Alsunaidi A, *et al.* Student-Led discharge counseling program for High-Risk medications in a teaching hospital in Saudi Arabia: A pilot study. *Saudi Pharm J*. 2021;29:1129–36.

30 DeSancho M, Munn JE, Billett HH, *et al.* Transition of care for pediatric and adult patients with venous thromboembolism: A National Quality Improvement Project from the American Thrombosis and Hemostasis Network (ATHN). *Thromb Res*. 2021;200:23–9.

31 Gurwitz JH, Kapoor A, Garber L, *et al.* Effect of a Multifaceted Clinical Pharmacist Intervention on Medication Safety after Hospitalization in Persons Prescribed High-risk Medications: A Randomized Clinical Trial. *JAMA Intern Med*. 2021;181:610–8.

32 Kane-Gill SL, Wong A, Culley CM, *et al.* Transforming the Medication Regimen Review Process Using Telemedicine to Prevent Adverse Events. *J Am Geriatr Soc*. 2021;69:530–8.

33 Magny-Normilus C, Nolido NV, Borges JC, *et al.* Effects of an Intensive Discharge Intervention on Medication Adherence, Glycemic Control, and Readmission Rates in Patients With Type 2 Diabetes. *J Patient Saf*. 2021;17:73–80.

34 Zabrosky R, Rubin EC, Liu E, *et al.* Improving Transitions-of-Care for Patients Discharged on High-Risk Antimicrobial Therapy. *Open Forum Infectious Diseases*. Netherlands 2021:310–1.

35 Lázaro Cebas A, Caro Teller JM, García Muñoz C, *et al.* Intervention by a clinical pharmacist carried out at discharge of elderly patients admitted to the internal medicine department: influence on readmissions and costs. *BMC Health Serv Res*. 2022;22:1–9.
